# Changes in firearm intentions and behaviors after the 2024 United States presidential election

**DOI:** 10.1186/s40621-026-00654-9

**Published:** 2026-01-30

**Authors:** Michael D. Anestis, Allison E. Bond, Kimberly C. Burke, Sultan Altikriti, Daniel C. Semenza

**Affiliations:** 1 New Jersey Gun Violence Research Center, Rutgers University, Piscataway, NJ USA; 2 Department of Urban-Global Public Health, Rutgers University, Piscataway, NJ USA; 3 Department of Sociology, Anthropology, and Criminal Justice, Rutgers University-Camden, Camden, NJ USA

## Abstract

**Background:**

Firearm purchasing patterns, intentions, and behaviors change over time in response to specific events. Additionally, the nature of these changes may be evolving over time or differ depending on the nature of the event in question. Given the intensity of the rhetoric surrounding gun violence leading up to the 2024 election, we sought to examine the extent to which firearm purchasing patterns, intentions, and behaviors changed following the 2024 Presidential election and the extent to which any such changes varied by population.

**Methods:**

A nationally representative sample was recruited to complete an online survey October 22-November 3, 2024 (*n* = 1,530) and assessed again January 7-January 22, 2025 (*n* = 1,359).

**Results:**

Identifying as Black was associated with increases in urges to carry firearms because of the election results (β = 0.16; 95%CI = 0.07-0.61). Liberal beliefs were associated with greater increases in urges to carry firearms because of the election results (β = 0.11; 95%CI = 0.01-0.13) and greater odds of storing firearms more quickly accessible because of the election results (OR = 2.11; 95%CI = 1.29–3.44).

**Conclusions:**

Individuals threatened by Trump administration policies appear to be experiencing urges to acquire firearms, carry them, and store them readily accessible. These results highlight that the current political environment may be fostering community-level decision making that, while motivated by the drive for protection, increases the risk for harm. Policies and programs that encourage secure storage and discourage firearm carrying may be increasingly important for the prevention of injury and death.

## Introduction

Firearm purchasing patterns change over time in response to specific events. For instance, purchasing surges frequently follow high-profile mass shootings and precede elections where progressive candidates may make electoral gains [1–3]. Researchers have posited that this pattern indicates efforts to acquire firearms in anticipation of potential policy changes that would restrict future firearm access [4]. In early 2020, an unprecedented surge in firearm sales corresponded with the onset of the COVID-19 pandemic [5], the increased strength of the racial justice movement in response to several murders of Black citizens by law enforcement officers, and the lead-up to the contentious 2020 Presidential election. Notably, the firearm purchasing patterns during this surge differed meaningfully from historical trends, with 50% of new firearm owners identifying as female and 20% as Black [6]. Additionally, multiple studies demonstrated that, in contrast to typical findings, those who purchased firearms during that surge – particularly first-time firearm purchasers – were more likely than anyone else to endorse lifetime or recent suicidal thoughts [7, 8].

This shift aligns with broader evidence that gun ownership patterns respond to presidential party, with Democrats more likely to own firearms under Republican presidents [9] and more open to future ownership than Independents [10]. While partisanship is more salient than political ideology in shaping gun attitudes [11, 12], gun owner identity, which is tied to conservative politics, is distinct from gun ownership itself [13] suggesting a need to examine factors beyond party affiliation in gun purchasing intentions. Liberal gun organizations have grown substantially during Republican administrations [14], and qualitative research suggests that Trump’s first presidency motivated firearm acquisition among Black Americans who perceived his administration as emboldening racial violence [15]. Neither of these patterns is reducible to partisan affiliation, motivating examination of how both political ideology and race independently shape firearm intentions and behaviors in response to elections.

Taken together, these findings highlight that major events can influence intentions and behaviors related to firearms. Additionally, the nature of these changes may be evolving over time or differ depending on the nature of the event in question. Several important questions remain unanswered, however. Prior research has focused almost exclusively on firearm purchasing patterns, without any assessment of related outcomes (e.g. carrying, storage). Additionally, relevant prior research has not examined the extent to which individuals report their intentions and behaviors being directly influenced by specific events or – more importantly – assessed individuals immediately prior to and following important events to capture change prospectively.

In response to this gap and given the intensity of the rhetoric surrounding gun violence leading up to the 2024 election [16], we recruited a nationally representative sample of adults immediately prior to and in the weeks following the election to assess the extent to which individuals report changes in firearm-related intentions and behaviors. We also assessed the extent to which individuals report that any such changes were directly impacted by the results of the election. Furthermore, we sought to characterize patterns in the identities and worldviews of those individuals who reported their firearm intentions and beliefs were directly impacted by election results. Findings from this study will provide novel information regarding firearm intent, acquisition, and storage habits surrounding the 2024 election, which can inform prevention efforts.

## Methods

### Procedures

Data collection was approved by the Rutgers Health Institutional Review Board (Pro2024001991). Electronic consent was obtained at baseline and reporting aligns with STROBE guidelines. The same set of participants completed the baseline survey in the two weeks leading up to the 2024 presidential election and the follow-up survey in the opening weeks of 2025. Baseline data were collected via Ipsos’ nationally representative probability-based KnowledgePanel October 22-November 3, 2024 (completion rate = 62%; final *n* = 1,530). Follow-up data were collected January 7-January 22, 2025 (completion rate = 90%; final *n* = 1,359).

Data weighting pre-election occurred through two steps. First, design weights for all KnowledgePanel assignees were computed to reflect their selection probabilities. Second, the design weights were raked to geodemographic distributions of the 18 and older US population, with benchmarks obtained via the 2024 March Supplement of the Current Population Survey. The resulting weights were trimmed and scaled to add up to the total number of respondents. For post-election weighting, starting with the pre-election weights, respondents were raked to geodemographic distributions of the 18 and older US population, obtained from the 2024 March Supplement of the Current Population Survey and PID benchmarks were obtained from the 2024 Pew’s National Public Opinion Reference Survey. The resulting weights were trimmed and scaled to add up to the total number of respondents.

## Measures

### Variables assessed at baseline

#### Intent to purchase firearms

Participants were asked “Are you planning to acquire a firearm in the next 12 months?” Answer choices included “Yes,” “No,” “Haven’t decided yet,” and “Prefer not to answer.”

#### Views on crime

Participants were asked “How much of a problem do you think crime is in this country?” Answer choices included “Not a problem at all,” “A small problem,” “A moderately big problem,” and “A very big problem.” Scores ranged from 0 to 3, with higher scores indicating views that crime is a bigger problem.

#### Perceived threat to democracy

Participants were asked “When thinking about democracy in the United States these days, do you believe…” with answer choices including the following: “There is a serious threat to our democracy,” “There may be a threat to our democracy, but it is not serious,” and “There is no threat to our democracy.” This item was derived from Wintemute et al. [17] and answers were coded 0 to 2, with higher scores indicating lower perceived threat to US democracy.

#### Support for political violence

Participants were presented with the following instructions: “Your view on the use of force or violence to advance an important political objective might depend on the specific objective that was involved. What do you think about the use of force or violence in the following situations?” Answer choices included “To return Donald Trump to the presidency this year,” “To stop an election from being stolen,” “To stop people who do not share my beliefs from voting,” “To prevent discrimination based on race or ethnicity,” “To preserve an American way of life based on Western European traditions,” “To oppose the government when it does not share my beliefs,” “and “To oppose the government when it tries to take private land for public purposes.” For each option, answer choices included “Never justified,” “Sometimes justified,” “Usually justified” and “Always justified,” with each item scored 1–4 and higher scores indicating greater support for political violence in that specific instance. Sum scores across items were utilized as a measure of overall support for political violence. Items were derived from [17].

### Variables assessed at Follow-Up

#### Intent to purchase firearms

The same item utilized at baseline was again administered at follow-up.

#### Firearm purchasing since the election

Participants were asked “Have you purchased a firearm since Election Day 2024?” Answer choices included “Yes,” “No,” and “Prefer not to Answer.”

#### Changes in urge to carry firearms due to election results

Participants who endorsed current household firearm access were asked “What impact did the 2024 election have on your urge to carry your firearm outside the home?” Answer choices included “The election results made me feel much less of an urge to carry my firearm outside my home,” “The election results made me feel somewhat less of an urge to carry my firearm outside my home,” “The election results had no impact on my urge to carry my firearm outside my home,” “The election results made me feel somewhat more of an urge to carry my firearm outside my home,” and “The election results made me feel much more of an urge to carry my firearm outside my home.”

#### Changes in firearm storage practices due to election results

Participants who endorsed current household firearm access were asked “Have you changed the way you store any of your firearms in response to the results of the 2024 Presidential election?” Answer choices included “No. I have not changed how I store any of my firearms since the election,” “No. I have changed how I store one or more firearms, but not because of the results of the election,” “Yes. I now store one or more firearms so that I can get to them more quickly,” and “Yes. I now store one or more firearms so that they are not as quick and easy to access.”

### Data analytic plan

To examine the association between demographic factors, political worldviews, and changes in the urge to carry firearms due to the results of the 2024 election, we utilized linear regression. To examine the association between demographic factors, political worldviews, and changes in firearm storage practices due to the results of the 2024 election, we utilized binary logistic regression. In each of these analyses, age, sex, Black non-Hispanic, White non-Hispanic, Hispanic, education, annual household income, views on how big of a problem crime is nationally, perceived level of threat to US democracy, support for political violence, and political beliefs served as independent variables.

## Results

### Firearm purchasing post-election

Only 26 individuals purchased firearms between baseline and follow-up (Table 1). As such, we were not statistically powered to examine between-group differences. Among those who purchased firearms during the study period, 40.7% were aged 45–59 (23.2% in full sample), 50% identified as female (50.0% in full sample), 20.0% identified as Black (12.1% in full sample), 30.7% identified as Hispanic (17.9% in full sample), and 14.9% identified as highly liberal (8.8% in full sample). Pre-election, 35.2% had indicated they did not intend to purchase a firearm in the next 12 months, 32.2% indicated they did intend to do so, and 32.6% indicated they were undecided (71.3%, 8.8%, and 18.0% in the full sample).

**Table 1:** Descriptive statistics for the entire sample, those who purchased firearms between baseline and follow-up, and those with varying levels of intent to purchase firearms within the next 12 months (assessed at follow-up; January 2025). Chi-Squared analyses and analyses of variance examining differences between purchase intent groups

	Full Sample	Purchase Since Election	Intent to Purchase Firearms in Next 12 Months (January 2025)				
			No	Yes	Have Not Decided	Prefer Not to Answer	
Sample Size	1,359	26	987	99	322	15	
	% (n)	% (n)	% (n)	% (n)	% (n)	% (n)	
Age							Χ^2^ = 21.35, *p* = .011, V = 0.07
* 18–29*	19.8 (269)	10.6 (3)	19.1 (175)_a_	22.6 (21)_a_	18.8 (56)_a_	35.7 (5)_a_	
* 30–44*	26.1 (354)	21.2 (6)	24.6 (225)_a_	30.1 (28)_a_	29.5 (88)_a_	7.1 (1)_a_	
* 45–59*	23.2 (315)	40.7 (11)	21.9 (200)_a_	28.0 (26)_a_	26.2 (78)_a_	35.7 (5)_a_	
* 60+*	30.9 (421)	27.5 (7)	34.4 (314)_a_	19.4 (18)_b_	25.5 (76)_b_	21.4 (3)_ab_	
Sex							Χ^2^ = 42.79, *p* < .001, V = 0.18
* Male*	48.9 (664)	50.0 (13)	43.0 (394)_a_	66.7 (62)_b_	61.3 (182)_b_	53.8 (7)_ab_	
* Female*	51.1 (695)	50.0 (13)	57.0 (522)_a_	33.3 (31)_b_	38.7 (115)_b_	46.2 (6)_ab_	
Race/Ethnicity							
* White*,* NH*	60.8 (826)	47.7 (12)	63.3 (580)_a_	47.3 (44)_b_	53.4 (159)_b_	64.3 (9)_ab_	Χ^2^ = 25.97, *p* < .001, V = 0.22
* Black*,* NH*	12.1 (164)	20.0 (5)	9.6 (88)_a_	21.7 (20)_b_	17.2 (51)_b_	21.4 (3)_ab_	Χ^2^ = 21.40, *p* < .001, V = 0.13
* Hispanic*	17.9 (243)	30.7 (8)	17.0 (156)_ab_	23.7 (22)_b_	21.2 (63)_ab_	0.0 (0)_a_	Χ^2^ = 7.60, *p* = .055, V = 0.08
Political Beliefs							Χ^2^ = 35.58, *p* < .001, V = 0.10
* Highly Conservative*	9.0 (123)	10.2 (3)	7.4 (66)_a_	15.2 (14)_b_	11.5 (34)_b_	6.7 (1)_ab_	
* Somewhat Conservative*	21.9 (298)	37.5 (10)	22.4 (199)_a_	25.0 (23)_a_	22.6 (67)_a_	33.3 (5)_a_	
* Moderate*	41.0 (557)	33.9 (9)	40.2 (358)_a_	42.4 (39)_ab_	47.3 (140)_b_	60.0 (9)_ab_	
* Somewhat Liberal*	16.4 (222)	3.6 (1)	19.9 (177)_a_	6.5 (6)_b_	12.8 (38)_b_	0.0 (0)_ab_	
* Highly Liberal*	8.8 (119)	14.9 (4)	10.1 (90)_a_	10.9 (10)_ab_	5.7 (17)_b_	0.0 (0)_ab_	
Intent to Purchase Pre-Election							Χ^2^ = 563.34, *p* < .001, V = 0.38
* No*	71.3 (969)	35.2 (9)	88.3 (808)_a_	19.6 (18)_b_	41.9 (34)_c_	38.5 (4)_bc_	
* Yes*	8.8 (119)	32.2 (8)	2.7 (25)_a_	55.4 (51)_b_	11.5 (34)_c_	30.8 (4)_b_	
* Have Not Decided*	18.0 (245)	32.6 (8)	8.2 (75)_a_	23.9 (22)_b_	45.3 (134)_c_	30.8 (4)_bc_	
* Prefer Not to Answer*	1.4 (20)	0.0 (0)	0.8 (7)_a_	1.1 (1)_a_	1.4 (4)_a_	0.0 (0)_a_	
	m (SD)	m (SD)	m (SD)	m (SD)	m (SD)	m (SD)	
Perception of Crime as a National Problem	3.22 (0.71)	3.09 (0.94)	3.16 (0.72)_a_	3.33 (0.81)_ab_	3.32 (0.63)_b_	3.40 (0.91)_ab_	F = 5.87, *p* < .001, _p_η^2^ = 0.01
Perceived Threat to Democracy	1.46 (0.68)	1.34 (0.66)	1.42 (0.66)_a_	1.61 (0.81)_a_	1.49 (0.69)_a_	1.60 (0.63)_a_	F = 2.84, *p* = .037, _p_η^2^ = 0.01
Support for Political Violence	9.82 (3.58)	10.82 (3.54)	9.52 (3.44)_a_	11.19 (3.91)_b_	9.81 (3.39)_a_	11.00 (3.76)_ab_	F = 7.86, *p* < .001, _p_η^2^ = 0.02

Cells within rows that do not share subscripts differ from one another at the *p* < .05 level in our analysis examining differences in the intent to purchase firearms following the election. Political Beliefs scored such that higher scores indicate more liberal beliefs; Perceptions of crime scored such that 1 = not a problem at all, 2 = a small problem, 3 = a moderately big problem, 4 = a very big problem; Perceived threat to democracy scored such that 1 = there is a serious threat to democracy, 2 = there may be a threat to our democracy, but it is not serious, 3 = there is no threat to our democracy; Support for political violence is scored such that a higher score indicates greater support for political violence across contexts

### Intentions to purchase firearms Post-Election

Those aged 60 and above represented a greater percentage of those not intending to purchase firearms post-election (34.4%) relative to those intending to purchase (19.4%) or undecided about doing so (25.5%; Χ^2^ = 21.35; *p* = .011; V = 0.07; Table 1).

Men represented a lower percentage of those not intending to purchase firearms post-election (43.0%) relative to those intending to (66.7%) or undecided about doing so (61.3%; Χ^2^ = 42.79; *p* < .001; V = 0.18).

White adults represented a higher percentage of those not intending to purchase firearms post-election (63.3%) relative to those intending to (47.3%) or undecided about doing so (53.4%; Χ^2^ = 25.97; *p* < .001; V = 0.22). Black adults represented a higher percentage of individuals planning to (21.7%) purchase firearms and undecided about purchasing firearms post-election (17.2%) relative to those planning not to (9.6%; Χ^2^ = 21.40; *p* < .001; V = 0.13).

Significant differences also emerged with respect to political beliefs (Χ^2^ = 35.58; *p* < .001; V = 0.10). Highly conservative individuals represented a lower percentage of those not intending to purchase firearms post-election (7.4%) relative to those intending to (15.2%) and those undecided about doing so (11.5%). Moderate individuals represented a lower percentage of those not intending to purchase firearms post-election (40.2%) than those undecided about doing so (47.3%). Somewhat liberal individuals represented a higher percentage of those not intending to purchase firearms post-election (19.9%) relative to those intending to (6.5%) and those undecided about doing so (12.8%). Among highly liberal individuals, similar percentages were observed in those not intending to purchase firearms (10.1%) and those intending to purchase (10.9%), while a lower percentage was found among those undecided (5.7%).

Significant differences emerged based on pre-election intent to purchase firearms (Χ^2^ = 563.34; *p* < .001; V = 0.38). Among individuals planning to purchase firearms at follow-up, 18.6% had indicated they were not planning to do so in the next 12 months when asked pre-election. Among those individuals, 33.3% had indicated pre-election that a quick Trump victory would increase their purchasing intent and 33.3% identified as highly liberal.

Lastly, significant differences emerged across multiple measures of worldviews. Specifically, individuals undecided about purchasing firearms post-election indicated crime is a bigger problem in the US (m = 3.32, SD = 0.63) relative to those not intending to do so (m = 3.16, SD = 0.72; F = 5.87; *p* < .001; _p_η^2^ = 0.01). Additionally, those intending to purchase firearms post-election endorsed higher levels of support for political violence (m = 11.19, SD = 3.91) than did those not intending to (m = 9.52, SD = 3.44) and those undecided about doing so (m = 9.81, SD = 3.39).

### Changes in urge to carry firearms Post-Election

Identifying as Black (β = 0.16; 95%CI = 0.07-0.61; Table 2) or female (vs. male; β = 0.10; 95%CI = 0.02-0.24), and endorsing more education (β = 0.13; 95%CI = 0.03-0.15) and more liberal political beliefs (β = 0.11; 95%CI = 0.01-0.13) were significantly associated with indicating the results of the 2024 election increased participants’ urge to carry firearms. Stronger views that crime a big problem (β=-0.12; 95%CI=-0.20–0.03), and perceiving a lesser threat to democracy (β=-0.11; 95%CI=-0.19–0.02) were associated with indicating that the results of the 2024 election decreased participants’ urge to carry firearms. Results are presented in Table 2.

**Table 2:** Linear regression examining correlates of changes in urges to carry firearms directly in response to the 2024 presidential election results

DV: Changes in urge to carry firearms directly in response to 2024 Presidential election results	β (95% CI)	*p*
Age	0.008 (−0.003 − 0.004)	0.862
Sex (ref: male)	0.097 (0.016 − 0.240)	0.025
Black, Non-Hispanic	0.156 (0.073 − 0.614)	0.013
White, Non-Hispanic	− 0.016 (−0.238 − 0.192)	0.833
Hispanic	0.008 (−0.237 − 0.265)	0.912
Education	0.130 (0.026 − 0.154)	0.006
Household Income	− 0.010 (−0.041–0.33)	0.833
Political Beliefs	0.106 (0.009 − 0.125)	0.024
Perception of Crime as a National Problem	− 0.120 (−0.196 - − 0.030)	0.008
Perceived Threat to Democracy	− 0.107 (−0.187 - − 0.020)	0.016
Support for Political Violence	− 0.085 (−0.031 − 0.000)	0.051

Political Beliefs scored such that higher scores indicate more liberal beliefs; Perceptions of crime scored such that 1 = not a problem at all, 2 = a small problem, 3 = a moderately big problem, 4 = a very big problem; Perceived threat to democracy scored such that 1 = there is a serious threat to democracy, 2 = there may be a threat to our democracy, but it is not serious, 3 = there is no threat to our democracy; Support for political violence is scored such that a higher score indicates greater support for political violence across contexts

### Changes in firearm storage Post-Election

Higher age (OR = 1.03; 95%CI = 1.00-1.06) was associated with greater odds of having made firearms more quickly accessible because of the 2024 election results relative to not having made any changes to firearm storage, whereas higher education was associated with lower odds of having done so (OR = 0.51; 95%CI = 0.29–0.89).

Lower perceived threat to democracy was associated with lower odds of making firearms more quickly accessible because of the 2024 election results (OR = 0.09, 95%CI = 0.01–0.68) and greater odds of making firearms less quickly accessible because of the 2024 election results (OR = 3.92, 95%CI = 1.03–14.96) relative to having not made any changes to their firearm storage.

Greater levels of support for political violence were associated with greater odds of having made firearm storage changes unrelated to the 2024 election results (OR = 1.11, 95%CI = 1.04–1.19), having made firearms more quickly accessible because of the 2024 election results (OR = 1.15, 95%CI = 1.01–1.30), and having made firearms less quickly accessible because of the 2024 election results (OR = 1.65, 95%CI = 1.26–2.18) relative to not having made any changes to their firearm storage.

Increasingly liberal beliefs were associated with greater odds of having made firearms more accessible (OR = 2.11; 95%CI = 1.29–3.44) and less accessible (OR = 3.08; 95%CI = 1.18–8.07) relative to having made no storage changes. Results are presented in Table 3.

**Table 3:** Binary logistic regression examining correlates of changes in firearm storage practices directly in response to the 2024 presidential election results

Ref: No Change in Storage	DV: Changes in firearm storage practices directly in response to 2024 Presidential election results											
	Change Made – Not Impacted by Election				Firearms More Quickly Accessible				Firearms Less Quickly Accessible			
	Wald	OR	95% CI	P	Wald	OR	95% CI	p	Wald	OR	95% CI	p
Age	0.09	1.00	0.98–1.01	0.766	5.05	1.03	1.00-1.06	0.025	1.42	1.04	0.98–1.10	0.233
Sex	0.10	1.08	0.66–1.75	0.758	2.04	0.50	0.19–1.29	0.153	0.00	0.97	0.17–5.40	0.972
Black, Non-Hispanic	0.55	1.54	0.49–4.80	0.460	0.05	1.25	0.17–9.11	0.824	0.02	1.21	0.06–24.04	0.901
White, Non-Hispanic	0.01	1.06	0.41–2.78	0.905	0.32	0.61	0.11–3.43	0.572	3.13	0.06	0.00-1.35	0.077
Hispanic	0.37	1.42	0.46–4.42	0.544	0.17	1.48	0.23–9.69	0.683	2.42	8.39	0.58-122.26	0.120
Education	0.01	0.99	0.75–1.30	0.917	5.62	0.51	0.29–0.89	0.018	1.44	1.80	0.69–4.67	0.230
Household Income	2.66	0.88	0.75–1.03	0.103	0.44	1.11	0.82–1.51	0.580	1.40	0.68	0.36–1.29	0.237
Crime as Problem	0.90	1.20	0.82–1.75	0.344	1.00	1.44	0.70–2.97	0.318	2.50	3.04	0.77–12.04	0.114
Threat to Democracy	0.69	0.85	0.58–1.25	0.406	5.49	0.09	0.01–0.68	0.019	4.01	3.92	1.03–14.96	0.045
Political Violence	10.32	1.11	1.04–1.19	0.001	5.01	1.15	1.01–1.30	0.025	12.91	1.65	1.26–2.18	< 0.001
Political Beliefs	0.91	1.14	0.87–1.48	0.340	8.90	2.11	1.29–3.44	0.003	5.25	3.08	1.18–8.07	0.022

Political Beliefs scored such that higher scores indicate more liberal beliefs; Perceptions of crime scored such that 1 = not a problem at all, 2 = a small problem, 3 = a moderately big problem, 4 = a very big problem; Perceived threat to democracy scored such that 1 = there is a serious threat to democracy, 2 = there may be a threat to our democracy, but it is not serious, 3 = there is no threat to our democracy; Support for political violence is scored such that a higher score indicates greater support for political violence across contexts

## Discussion

Our findings – collected using a nationally representative sample of adults immediately prior to and following the 2024 election - indicate significant changes in firearm intentions and behaviors between November 2024 and January 2025. The changes emerged across three domains: intentions to purchase firearms, changes in the urge to carry firearms, and changes in firearm storage practices. Although there was some variability in the results, the overall pattern indicated that individuals who may be viewed as threatened by Trump administration policies (e.g., diversity related changes) and who may perceive a strong threat to democracy may be considering or making changes to firearm access directly in response to election results, likely as a method of increasing perceived security.

Black adults, in particular, exhibited a notable pattern of post-election firearm intentions and urges. Specifically, Black adults represented a higher percentage of those intending to purchase firearms post-election (21.7%) or undecided about whether to purchase a firearm (17.2%) relative to individuals not intending to purchase a firearm in the next 12 months (9.6%). This aligns with findings from the 2020–2021 purchasing surge and further supports the notion that the demographics of firearm ownership may be shifting relative to historical trends [18]. Additionally, identifying as Black was associated with greater increases in the urge to carry firearms directly in response to the results of the 2024 election. This result, in particular, highlights that members of the Black community may feel a greater need to protect themselves in the context of a second Trump administration. In a recent nationally representative sample of Black adults, self-protection was the most commonly endorsed reason for firearm carrying (88.3%), but a meaningful minority (14.9%) indicated that they carry due to lack of faith in police [19]. If Black adults feel that the election results will render their communities less safe through policies that impact community disadvantage, policing practices, or other factors relevant to safety outside the home, this would explain both the drive to acquire firearms and increased urges to carry specifically in response to election results. There are substantial public health implications to this particular result. Black men and boys have seen a meaningful increase in their suicide rate (overall and firearm-specific) [20], corresponding with the higher incidence of firearm ownership in the homes of Black adults [6]. Recent research has highlighted that firearm carrying is associated with gun violence exposure, particularly among those who identify as Black [21], and that gun violence exposure is associated with increased risk for suicidal thoughts and behaviors [22]. The drive to purchase and carry firearms among Black adults – a response in part to the results of the most recent election – may thus further contribute to alarming trends in suicide rates at a time when federal policies are discouraging work that aims to develop, test, and implement programs aimed specifically at addressing the needs of minoritized communities.

A parallel series of results emerged when considering political views, particularly with respect to those who endorsed highly liberal beliefs. Although they represented a small minority of both groups, highly liberal individuals comprised highly similar percentages of those who reported post-election that they do (10.9%) and do not (10.1%) intend to purchase firearms in the next 12 months. Additionally, increasingly liberal political beliefs were significantly associated with stronger increases in the urge to carry firearms directly in response to the election results. Finally, with each unit increase in the degree of liberal beliefs (e.g. from highly conservative to somewhat conservative, from moderate to somewhat liberal), individuals were 2.11 times more likely to endorse having made firearms more readily accessible as a result of the 2024 election relative to not having made any storage changes. Increasingly liberal beliefs were also associated with greater odds of having made firearms less readily accessible because of the election (OR = 3.08) relative to not having made any changes, indicating that the election prompted a bifurcated response across firearm owners with more liberal political beliefs. Across all three domains, highly liberal individuals appear to have experienced shifts in their intentions and behaviors related to firearms, with much of those changes described as being directly related to the election results. Similar to the findings among Black adults, this may indicate that individuals within communities threatened by the current political environment are changing their firearm practices to increase their sense of safety.

Our findings related to perceived level of threat to US democracy exhibited a similar pattern. Higher levels of perceived threat to US democracy were associated with greater increases in urges to carry firearms and odds of storing firearms more quickly accessible due to the election results and lower odds of storing firearms less quickly accessible because of the election results. Our item assessing perceived threat to democracy did not ask participants to specify the source or nature of the threat so interpretation is somewhat limited. That said, the results provide a fairly straightforward picture of individuals perceiving that the structure and stability of the United States are under threat and both experiencing changes in urges to carry and making changes to firearm storage practices directly because of election results and in a manner that increases the quick accessibility of firearms. Although we cannot conclusively demonstrate that the perceived threat is the current administration, the fact that reported changes were said to be a response to the election results renders that interpretation the most parsimonious option and again highlights that certain communities feel compelled to protect themselves in an environment they view as hostile to their way of life.

The final set of results focused on support for political violence and was somewhat more difficult to interpret. Those who reported post-election that they intend to purchase firearms in the next 12 months reported higher mean levels of support for political violence relative to those not intending to purchase and those undecided about doing so. It appears that support for political violence might be motivating individuals to acquire firearms, perhaps as a method of pushing back against perceived injustices. In contrast, however, greater support for political violence was associated with decreased urges to carry firearms in response to election results, which may indicate those who support political violence feel safer within the context of a Trump administration. Lastly, greater levels of support for political violence were associated with greater odds of making firearm storage changes unrelated to election results, storing firearms more quickly accessible because of the election results, and storing firearms less quickly accessible because of the election results. This contradictory pattern of findings has several plausible interpretations. One possibility is that greater support for political violence is associated with more erratic thoughts and behaviors related to firearms. Such individuals might be unsettled with respect to the environment around them and may make frequent and contradictory changes in their behaviors in response to daily events or shifting concerns about the future. This might also reflect dynamic changes between defensive (e.g. storage and purchasing) and offensive (carrying) behaviors. Alternatively, our aggregate total score of support for political violence, comprised of a variety of situations with varying levels and types of political leanings, might be too heterogenous, resulting in a combination of results difficult to interpret because of the structure of the variable itself. Future work might consider examining support for political violence in specific circumstances rather than combining contexts into a single variable.

This study had several noteworthy limitations, including the low incidence of firearm purchasing during the study period and the use of self-report data. Furthermore, political party affiliation was coded in such a manner that individuals not affiliated with a political party and those not willing to describe their party affiliation were scored identically, thereby preventing us from examining whether partisanship exhibits a similar effect within our model. Nonetheless, our findings provide a timely examination of how the 2024 presidential election impacted firearm intentions and behaviors and imply that groups threatened by the current administration are seeking more ready firearm access. Although firearms remain far more common within conservative communities, the outcome of the 2024 election may have prompted some liberal and minoritized communities to shift perspectives on firearms. Any such shift would require a related reconsideration of how we think about what prompts risk, where risk is most likely to emerge, and how best to reach specific groups of firearm owners to encourage safer practices. Regardless of an individual’s identity or political ideology, more frequent carrying and unsecure storage increases the risk for a host of problematic outcomes. The drive for protection is an understandable impulse, but if some communities are motivated by that drive to increase their risk of injury and death in pursuit of the perception of safety, it is vital that we work to diminish the fears driving those behaviors, disseminate messaging and programs that promote alternative methods to achieve the feeling of safety, and promote programs that can mitigate the harm associated with increased firearm access in these communities.

## Data Availability

The dataset used during the current study is available from the corresponding author upon reasonable request.
